# Research on the Top-Down Parsing Method for Context-Sensitive Graph Grammars

**DOI:** 10.1371/journal.pone.0142776

**Published:** 2015-11-30

**Authors:** Yi Wang, XiaoQin Zeng, Han Ding

**Affiliations:** 1 Institute of Intelligence Science and Technology, Hohai University, Nanjing, P.R. China; 2 School of Mathematical and Computer Sciences, Hubei University of Arts and Science, Xiangyang, China; Xiamen University, CHINA

## Abstract

The parsing problem is one of the key problems of graph grammars. The typical parsing algorithm uses the bottom-up method. The time-complexity of this method is high, and it is difficult to apply. In order to reduce the time-complexity, this paper uses the top-down method for parsing. This method avoids the subgraph isomorphism judgment and selects the productions specifically, so that the time-complexity is greatly reduced.

## Introduction

Graph grammar is used to define and analyze a graph [1–10]. The graph in the graph grammar is abstracted as a two-dimensional object with nodes and edges (the relationship between the nodes).

**Definition 1** G = (V, E, L_V_, L_E_, S and T) is a graph, among them:

V is a set of the nodes in graph G and consists of the terminal set V_T_ and the non-terminal set V_N_; E is a set of the edges; L_V_ is a set of the node labels; L_E_ is a set of the edge labels; and S: E →V, T: E →V are the mappings of the edges to the nodes, presenting the beginning and the end of each edge, respectively.

**Definition 2** A production is a rule that is written as g_l_: = g_r_; g_l_ and g_r_ are two graphs, respectively called the left side and the right side. Using a production, a given graph can be converted to another graph. That is to say, a sub-graph of the given graph that is isomorphic with g_l_ (g_r_) can be replaced with a graph that is isomorphic with g_r_ (g_l_).

**Definition 3** A graph that uses the productions for conversion is the host graph, written as g^host^. In addition, g_l_^host^ is a sub-graph of the ghost that is isomorphic with g_l_.

**Definition 4** From g^host^, removing all the nodes and edges that are connected with the nodes in the g_l_^host^ will give the Residual Graph, called g^residual^. In addition, an edge such that its two nodes are covered by the g_l_^host^ and the g^residual^, respectively, is called a dangling edge.

**Definition 5** A g_r_^host^ is a graph that is isomorphic with g_r_ and it will replace the g_l_^host^ in the process of the conversion.

In graph grammars, the productions are the basis of the conversion for the g^host^. However, when the g^host^ is converted, it needs to explain how to connect with the g^residual^ after the g_l_^host^ is replaced by g_r_^host^. Generally, it goes through the corresponding rules for the instructions. Such rules are referred to as **Embedding Rules**.

**Definition 6** A Graph Grammar gg contains three elements:

1. An initial graph;
2. A set of productions that are used for transformations;
3. A set of embedding rules.

**Definition 7** A language generated from a graph grammar is a graph and is deduced using the productions starting from the initial graph, and the graph does not contain an endpoint.

The operations for a graph grammar are divided into two categories [11]: derivation and parsing. The former works by looking for a sub-graph of the initial graph that is isomorphic with the left graph of the production and replacing that sub-graph with a graph that is isomorphic with the right graph of the production. Through the derivation, it can obtain the languages of the graph grammar. The latter category, parsing, is the opposite: the host graph is called the language of the graph grammar if it can obtain the initial symbol of the grammar.

The typical parsing algorithm [11] uses the bottom—up method. This method involves the subgraph isomorphism judgment (an N-P problem), and all the productions are used here, due to which, the time-complexity is higher. Zhang put forward a Selection-Free Parsing Algorithm (SFPA) based on RGG (Reserved Graph Grammar) [12]. The time-complexity of the algorithm is at the polynomial level, but the productions need to satisfy the selection-free condition, which is too strong a limitation.

This paper uses the top-down method for parsing. This method avoids the subgraph isomorphism judgment and selects the productions specifically, so the time-complexity is reduced greatly.

## Typical Context-Sensitive Graph Grammars

### Layered Graph Grammars (LGG)

**Fig 1:**
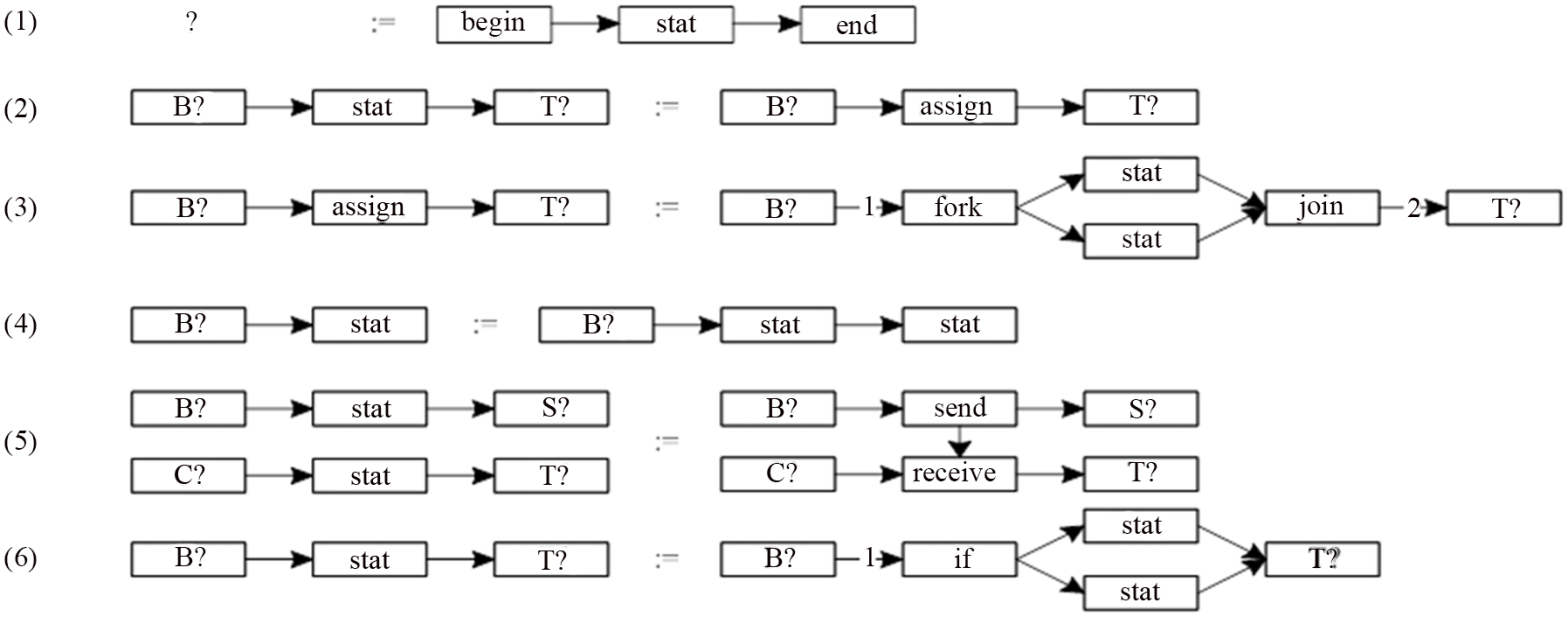
A set of productions of LGG.

In the productions of the LGG [13], the context information is the nodes, and the corresponding nodes have the same labels. That is to say, when embedding, these nodes will not be changed. As shown in Fig 1.

In Fig 1, a wildcard of “?” is introduced to the context nodes, that denotes a set of the labels. That is to say, as long as the context nodes meet the set, that can be identified as the corresponding nodes.

**Fig 2:**
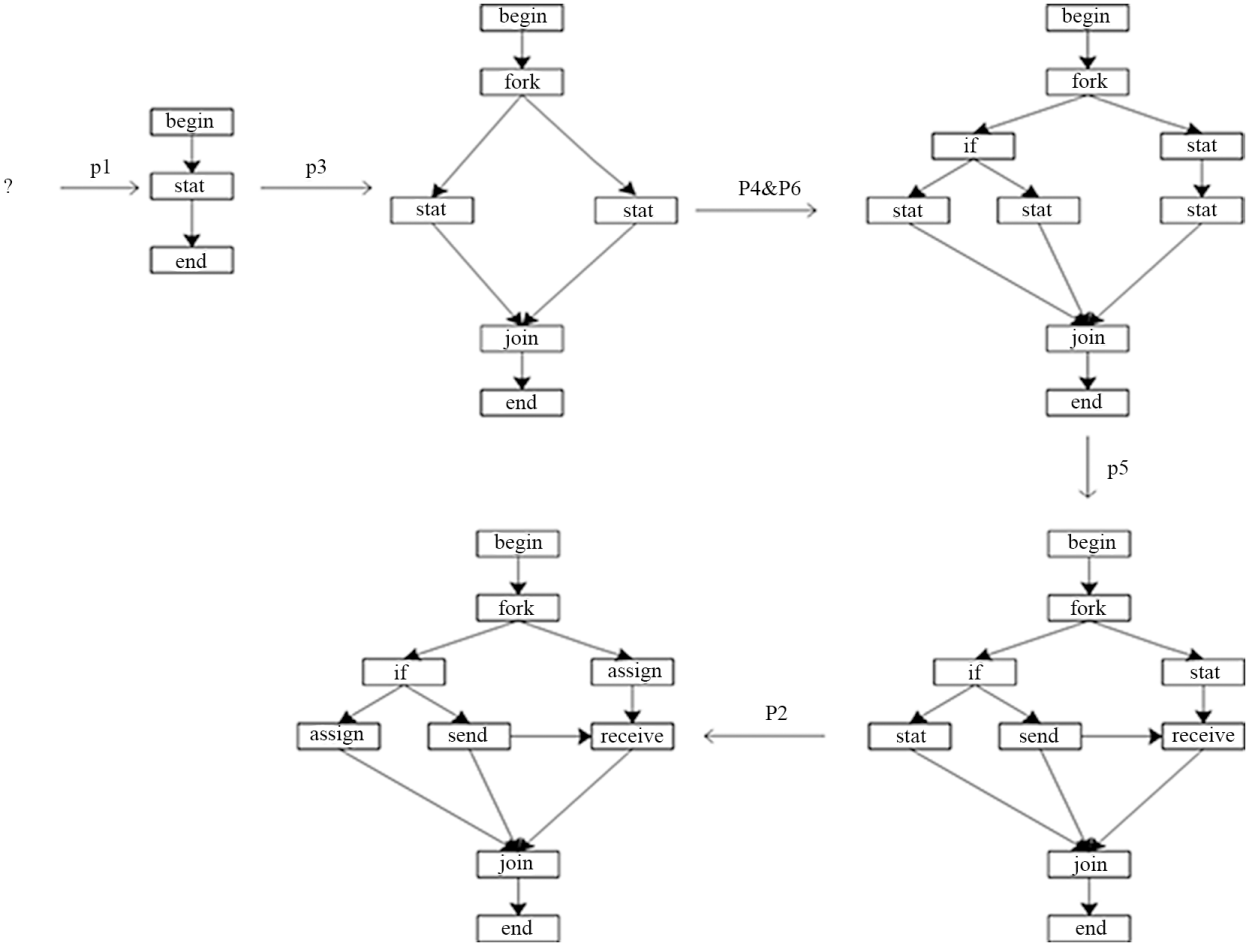
A derivation process of LGG.

On embedding method of LGG, the corresponding entity is the node both side of the productions, so these nodes are the context nodes. When embedding, through the context nodes connected to the residual graph and the nodes among the residual graph can not connect with other than the context nodes among the replacing graph. As shown in Fig 2.

### Reserved Graph Grammar (RGG)

**Fig 3:**
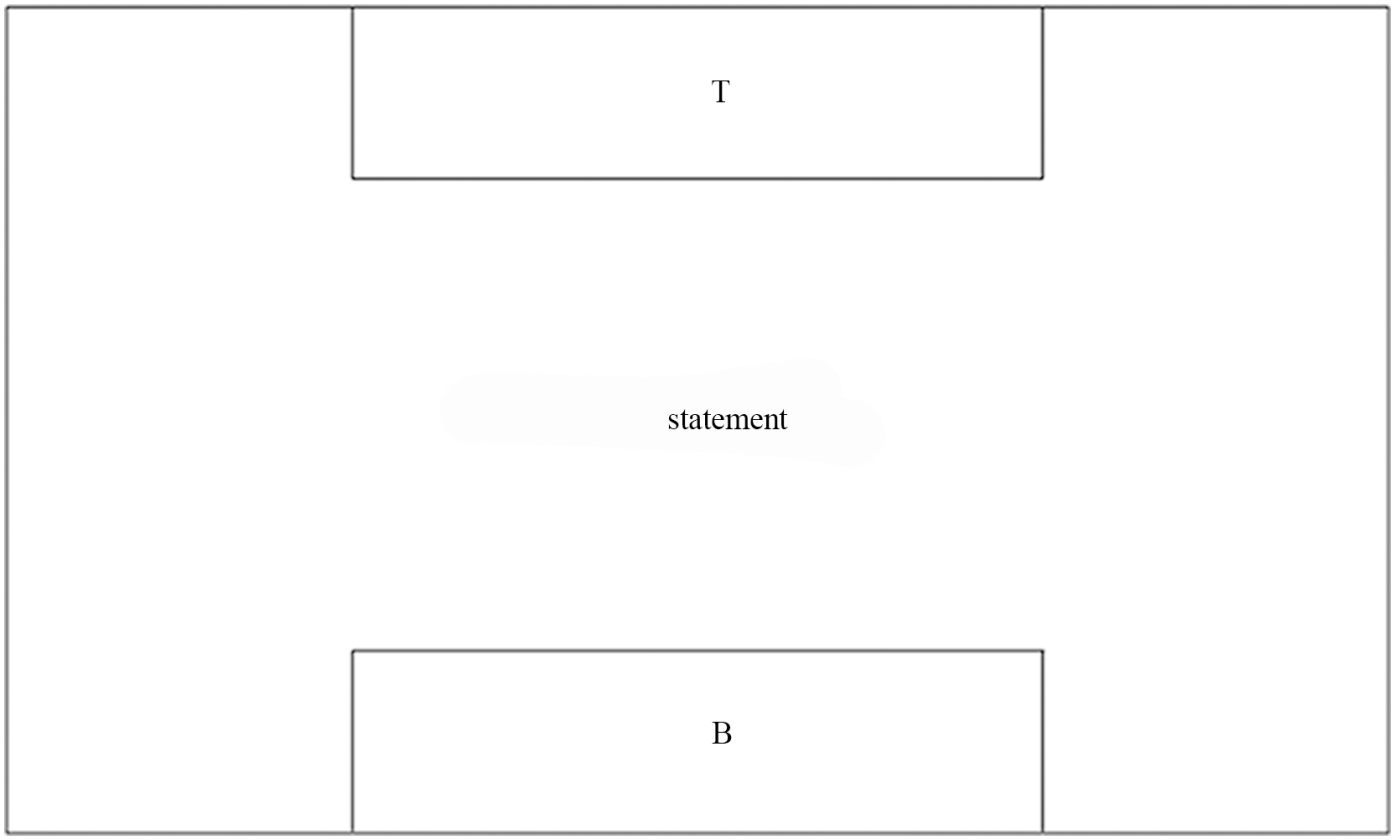
The node structure of RGG.

Zhang presents the RGG based on the LGG. In RGG, a node is a two layers structure, which includes a super vertex and some vertexes. As shown in Fig 3, statement is the label of a super vertex, T and B are the labels of two vertex. These super vertex and vertex are the terminal nodes of the edge in the graph.

On embedding method of RGG, a mark is assigned to the vertex. Through the corresponding relation between the nodes with the marks both side of the productions to complete the embedding process.

**Fig 4:**
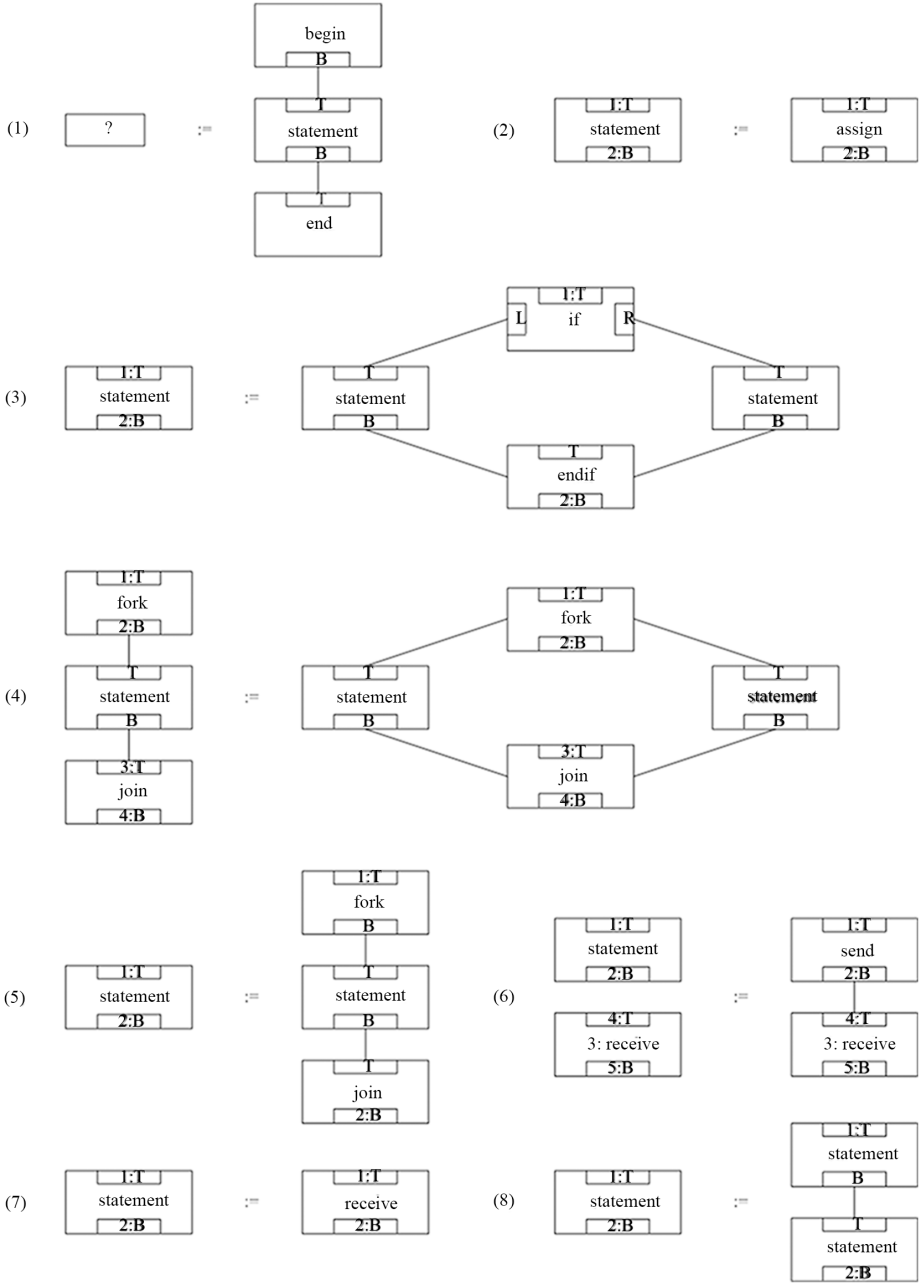
A set of productions of RGG.

As shown in Fig 4 is a set of productions of RGG.

**Fig 5:**
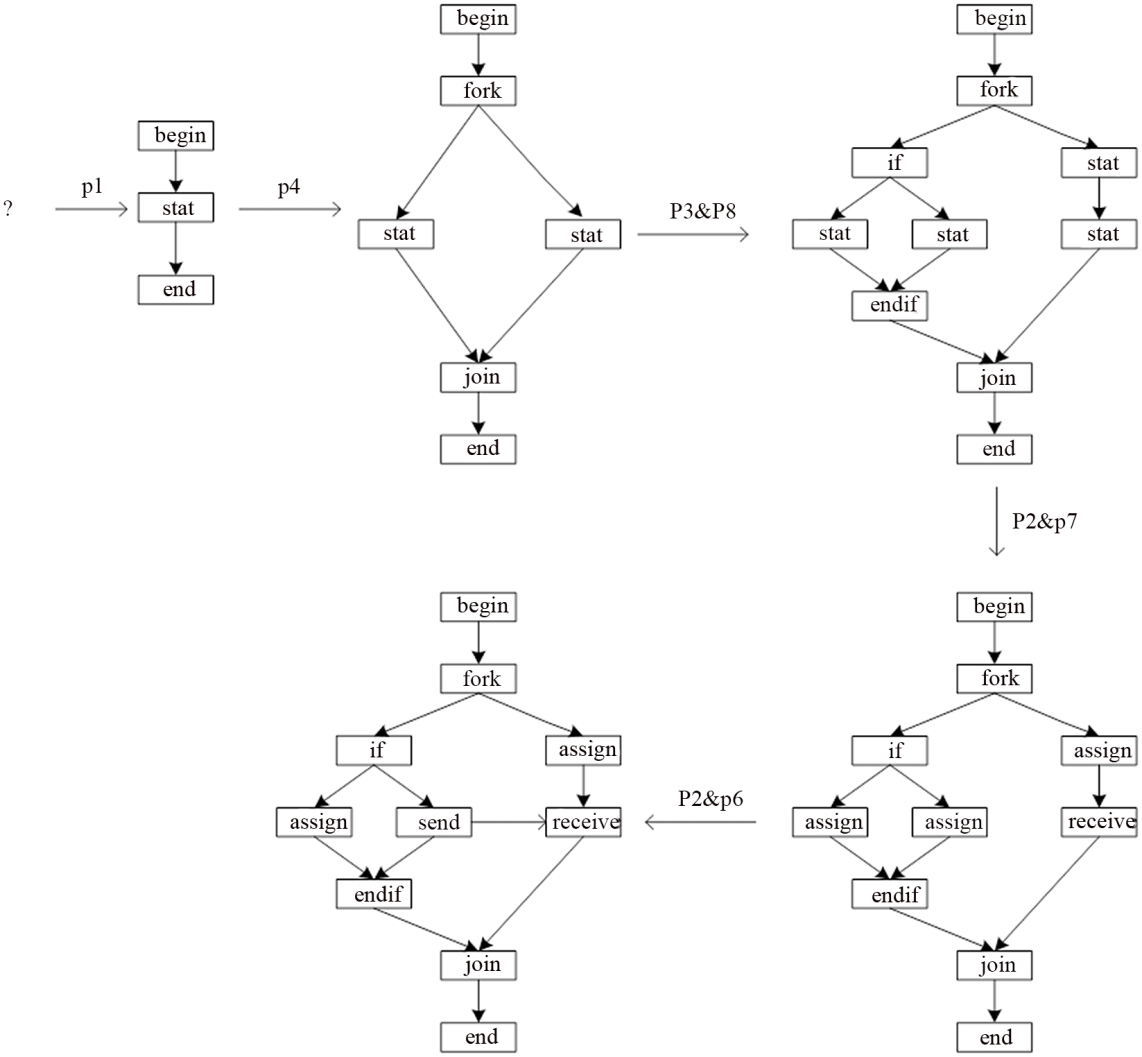
A derivation process of RGG.

As shown in Fig 5 is a derivation process of RGG.

Later, Kong put forward the SGG [14], its main characteristic is that joining the space information to the RGG, and extend its space processing ability. Zeng propose the EGG [15], its main characteristic is that the corresponding entity is the edge both side of the productions, through the corresponding relation between the edges to complete the embedding operation.

## Component-Based Graph Grammars

The existing context-sensitive graph grammars, such as Layered Graph Grammars (LGG), Reserved Graph Grammar (RGG) and Edge-Based Graph Grammar (EGG) [14], all use the nodes and the edges as the basic graph elements and complete the embedding operation through the corresponding relation of the left and the right graph elements. This paper presents a formalism of component-based graph grammar (CGG); it makes the nodes and the edges that connect with the nodes components and makes the edges that are a terminal in the graph the interface. Multiple components construct a union through the interface, which makes the unions the left-side and the right-side of a production. The difference between CGG and the exiting graph grammars is that when parsing, it makes the components the basic unit and uses the top-down parsing algorithm to match each component successively.

**Definition 8** A Component = (UpInterface, Node, DownInterface) is a triple, and among them, UpInterface and DownInterface is a set of the input interfaces and the output interface, respectively, collectively known as the interface.

**Definition 9** An InputInterface = {UI|S(UI) is not sure∧T(UI) = Node}, and an OutputInterface = {DI|S(DI) = Node∧T(DI) is not sure}. Among them, S is the start node function, and T is the end node function.

**Definition 10** The components have three styles: when UpInterface = Ø, the component is called the Up-Component; when DownInterface = Ø, the component is called the Down-Component; and when UpInterface = Ø and DownInterface = Ø, the component is called the ordinary component. In the case of no special instructions, the components are mentioned in this paper as the ordinary components.

**Definition 11** An Interface = (Label, Type, S, and T) is a quad, where Label is the label of the interface and Type is the interface type, and it has two styles: Up and Down.

When Type = Up, the interface is the UpInterface of the component, and S(1) is not sure and T(1) = Node; when Type = Down, the interface is the DownInterface, and T(1) is not sure and S(1) = Node.

**Fig 6:**
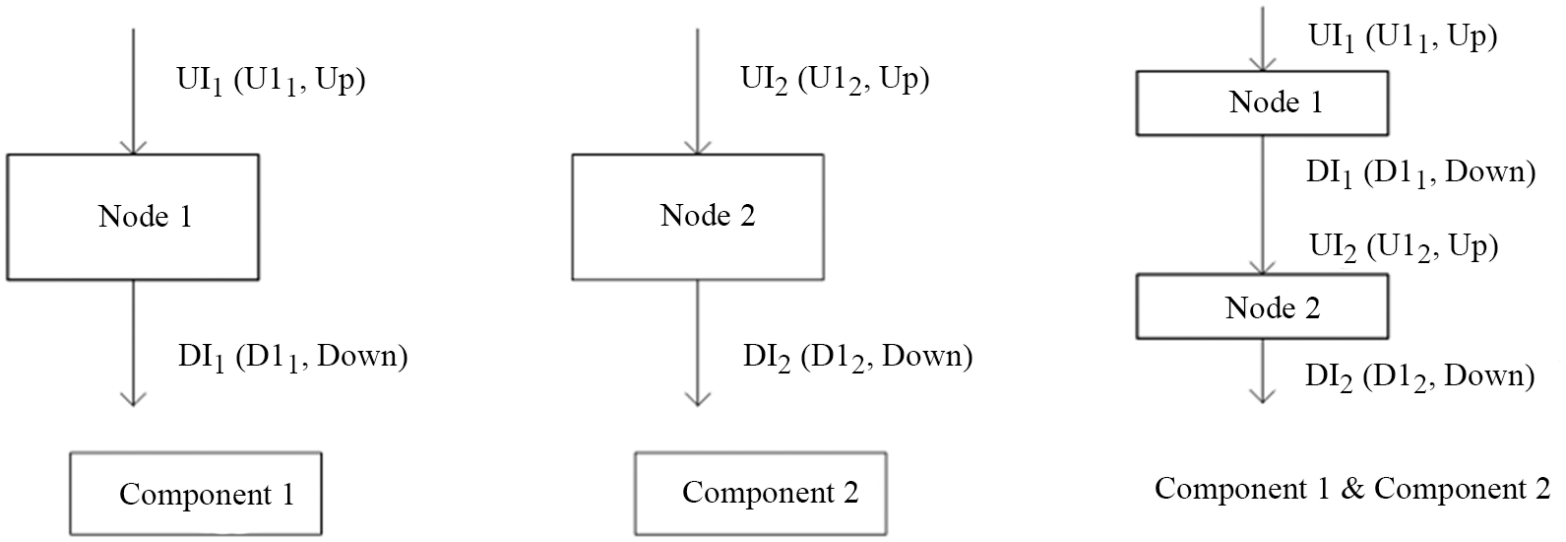
The connection of two components.

**Definition 12** Two components are connected with the interfaces, written as Component1&Component2. It is shown in Fig 6.

**Definition 13** A node = (Label, Up, Down) is a triple, and among them, Label is the label of the node; Up and Down are the input and the output interface, respectively.

**Definition 14** A Union = &(Component1,Component2, tComponentn) is composed of multiple components. Specifically, when there is only one component in a union, the union is a component; when some interfaces are not connected with the components, the union is called Open-Union. Otherwise, the union is called Closed-Union.

**Definition 15** A host graph of a graph grammar is a Closed-Union.

**Fig 7:**
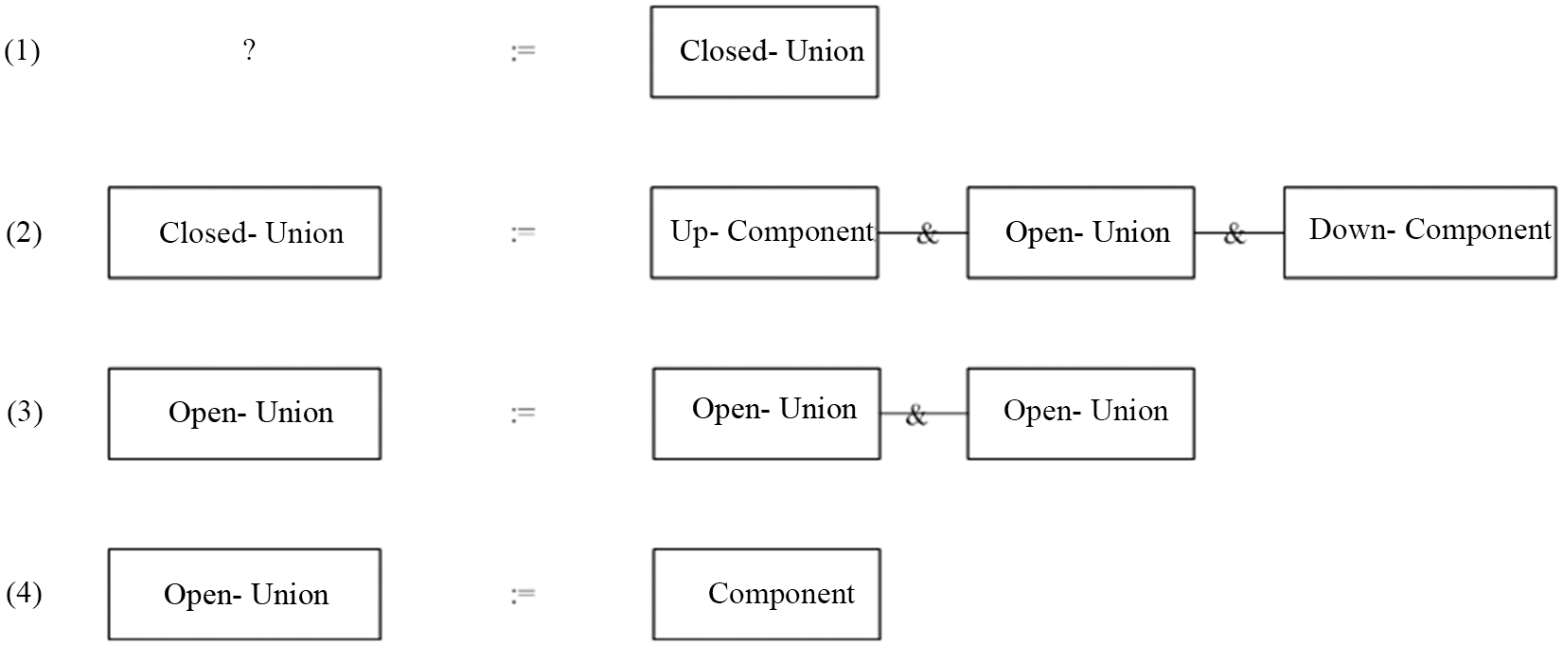
The styles of the productions of CGG.

**Definition 16** A production P: pL: = pR, pL and pR are two Open-Unions. Specifically, when the left-hand side of a production is the initial graph, the right-hand side is a Closed-Union. It is shown in Fig 7.

**Fig 8:**
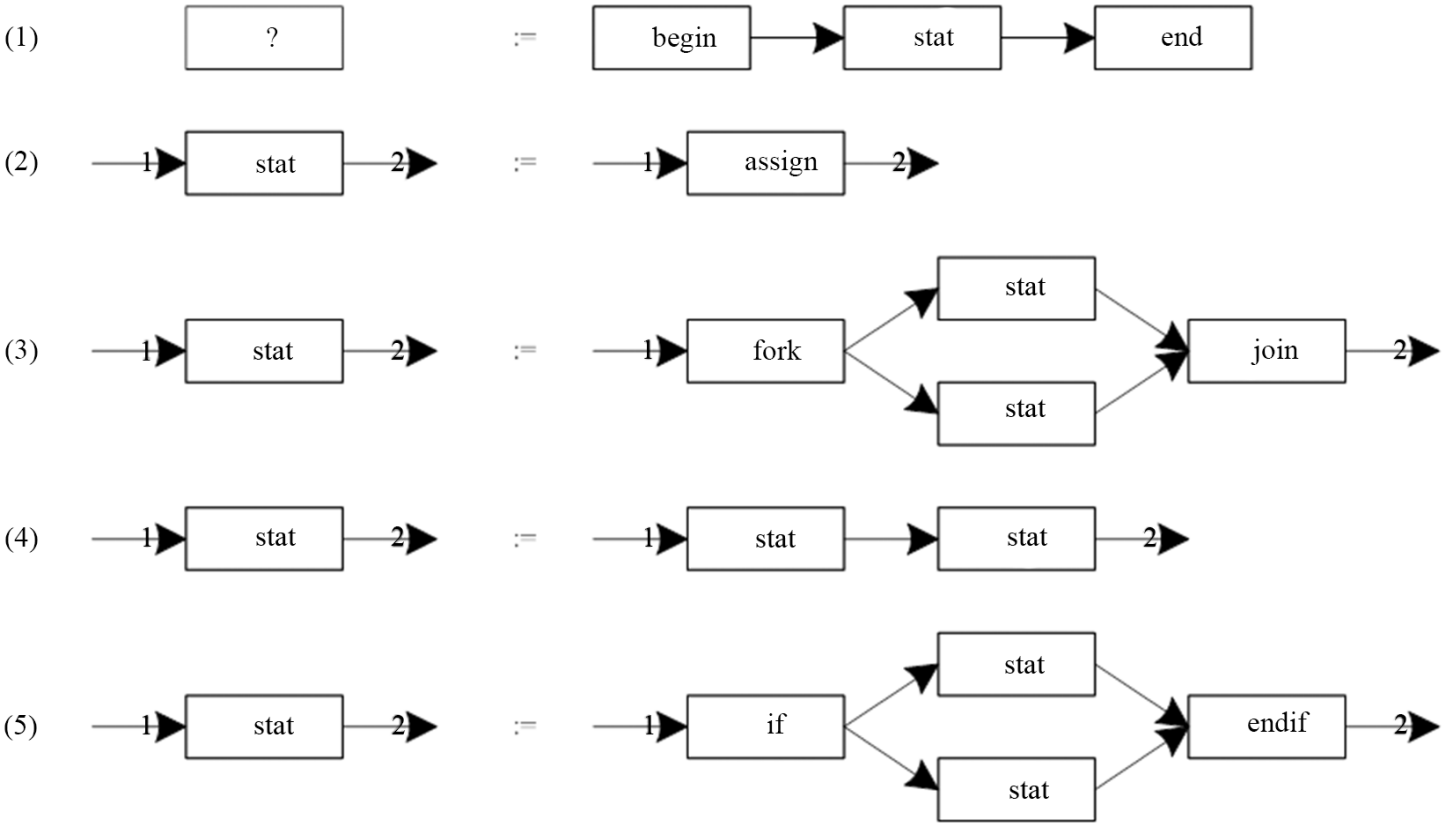
A set of productions of CGG.

The comparison of CGG and EGG:

(1) The nodes in CGG are equivalent to the nodes in EGG, and the interfaces in CGG are equivalent to the suspensions in EGG. Therefore, the production form of CGG is the same as that of EGG. Fig 8 shows a set of the productions of CGG.

**Fig 9:**
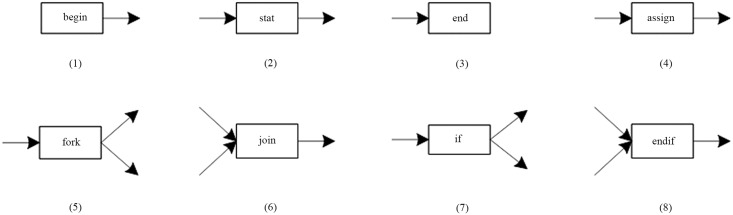
Components in Fig 8.

In Fig 8, there are the following components. Shown as Fig 9.

In Fig 9, (1) is an Up-Component, and (3) is a Down-Component; the rest of them are the ordinary components.

**Fig 10:**
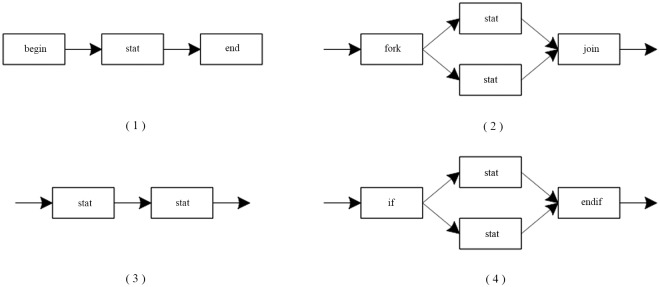
The unions in Fig 8.

In Fig 8, the unions as Fig 10:

In Fig 10, (1) is a Closed-Union, and the rest of them are the Open-Union.

(2) While embedding, for EGG, it completes the operation using the corresponding relation of the dangling edges of the left-hand side and the right-hand side of the productions; for CGG, it uses the corresponding relation of the interfaces.

(3) The biggest difference between CGG and EGG is that CGG makes the component the basic unit and uses the top-down method to parse.

## The Top-Down Parsing Algorithm

For graph grammars, how to judge whether a given graph is a language of that grammar is the key problem. It includes two aspects: it must ensure that the parsing process completes within the finite steps and that the time-complexity is not too large. Otherwise, it is not convenient for practical applications.

For the former aspect, we adopt the judgment method that is similar to EGG and make the components and the unions the terminals and the un-terminals, respectively. For the latter aspect, we assign a component as a basic element and adopt the top-down parsing algorithm to match each component in order. Because the algorithm avoids the subgraph isomorphism judgment and the production selection is also more targeted, the algorithm does not blindly put all the productions into the host graph to look for the isomorphism subgraph, and thus, the time-complexity will be greatly reduced.

The time-complexity of the existing parsing algorithm is higher for the following reasons:

1. It must judge the subgraph isomorphism, which is an N-P problem in the process of looking for the index;
2. It is necessary to go back repeatedly during the process of parsing.

Although the SFPA algorithm reduced the back times, its production should still meet the selection-free conditions. The judgment condition itself is also time-consuming, and the limitation on the production is too strong.

### Definition

**Definition 17** A node in CGG is defined as follows:

Node (Label, In, Out, DIn, DOut, Matched, matched), where

Label is the node label;

In is the in-degree of a node;

Out is the out-degree of a node;

DIn is the dangling edges in-degree of a node;

Dout is the dangling edges out-degree of a node;

Matched represents whether the two nodes matched success: if successful, the value is Y: otherwise, the value is N;

matched represents whether a node matched success: if successful, the value is Y: otherwise, the value is N.

**Definition 18** An interface of a component in CGG is defined as follow:

Interface (Label, Type, Dangling, Matched, matched), where

Label is the label of an interface;

Type is the type of an interface; the value is Up or Down;

Dangling represents whether an interface connects a component: if it connects, the value is Y; otherwise, the value is N;

Matched represents whether the two nodes matched success: if successful, the value is Y; otherwise, the value is N;

matched represents whether a node matched success: if successful, the value is Y; otherwise, the value is N.

In order to make it convenient to describe it, we mark an interface in an edge. Matching judgment for two components includes three aspects: up-interfaces, node and down-interfaces, corresponding to the in-edges, the node and the out-edges, respectively, in a general graph.

**Definition 19** A host graph G, a set of productions P,*p*_*m*_ ∈ *P*, *v*_*i*_ ∈ *G*,*v*_*j*_ ∈ *p*_*m*_, SetofHostNodeIn(*v*_*i*_) and SetofproNodeIn(*v*_*j*_) are the set of the in-edges of the nodes of *v*_*i*_ and *v*_*j*_, respectively. An in-edge *E*_*i*_ ∈ *SetofHostNodeIn*(*v*_*i*_) and an in-edge *E*_*i*_ ∈ *SetofproNodeIn*(*v*_*j*_). If(Label(E_i_) = Label(E_j_)) ∨ E_j_.*Dangling* = *Y*, then Matched(E_i_,E_j_) = Y, else Matched(E_i_,E_j_) = N.

**Definition 20** A host graph G, a set of productions P, *p*_*m*_ ∈ *P*, *v*_*i*_ ∈ *G*,*v*_*j*_ ∈ *p*_*m*_, if ∃*E*_*i*_(s(*E*_*i*_) = *v*_*i*_),∃*E*_*j*_(s(*E*_*j*_) = *v*_*j*_), then *Matched*(*E*_*i*,_ *E*_*j*_) = *Y* ⇔ (*E*_*i*_.Label = *E*_*j*_.Label) ∨ (*E*_*j*_.Dangling = Y),

if *Matched*(*E*_*i*,_ *E*_*j*_) = *Y*, then E_i_.matched = *Y* ∧ *E*_*j*_.*matched* = *Y*.

**Definition 21** A host graph G, a set of productions P, *p*_*m*_ ∈ *P*, *v*_*i*_ ∈ *G*,*v*_*j*_ ∈ *p*_*m*_, SetofHostNodeOut(*v*_*i*_) and SetofproNodeOut(*v*_*j*_) are the set of the out-edges of the nodes of *v*_*i*_ and *v*_*j*_, *E*′_*i*_ ∈ *SetofHostNodeOut*(*v*_*i*_) and *E*′_*j*_ ∈ *SetofproNodeOut*(*v*_*j*_) is an out-edge of the nodes of *v*_*i*_ and *v*_*j*_, then

$$\begin{array}{ll} Matched(v_{i},v_{j})=Y\Leftrightarrow & (v_{i}.Label=v_{j}.Label)\wedge \\ & (\forall E_{i}(\exists E_{j}(Matched(E_{i},E_{j})=Y)))\wedge \\ & (\forall E_{j}(\exists E_{i}(Matched(E_{i},E_{j})=Y)))\wedge \end{array}$$

$$(\forall E_{i}(E_{i}.matched=Y))\wedge$$

$$(\forall E\prime_{i}(\exists E\prime_{j}(Matched(E\prime_{i},E\prime_{j})=Y)))\wedge$$

$$\left(\forall E\prime_{j}(\exists E\prime_{i}(Matched(E\prime_{i},E\prime_{j})=Y))\right)$$

**Definition 22** A host graph G, a set of productions P. *p*_*m*_ ∈ *P*, *v*_*i*_ ∈ *G*,*v*_*j*_ ∈ *p*_*m*_. In order to compute the value of *v*_*i*_.matched, it needs to compute Matched(*v*_*i*_,*v*_*j*_). In this process, *p*_*m*_ is the target production, *v*_*i*_ is the current judgment node, and *v*_*j*_ is the target node of *v*_*i*_.

**Definition 23** A production *p*_*i*_ ∈ *P*, the formalism is PiL: = PiR. SiL = {${\vec{E}}_{iL}$, ${\overset{\leftarrow }{E}}_{iL}$}, SiR = {${\vec{E}}_{iR}$, ${\overset{\leftarrow }{E}}_{iR}$} are the set of the dangling edges of the left hand and the right hand sides of the production, and $e_{j}\in {\vec{E}}_{iL}$, and the node v = T(ej)is the above link node. SetofupNode(*p*_*iL*_) is the set of the above link nodes of the left hand side of a production of *p*_*i*_.

**Definition 24** A production *p*_*i*_ ∈ *P*, the formalism is PiL: = PiR. SiL = {${\vec{E}}_{iL}$, ${\overset{\leftarrow }{E}}_{iL}$}, SiR = {${\vec{E}}_{iR}$, ${\overset{\leftarrow }{E}}_{iR}$} are the set of the dangling edges of the left hand and the right hand sides of the production, and $e_{k}\in {\overset{\leftarrow }{E}}_{iL}$, the node v = S(ek)is the below link node. SetofdownNode(*p*_*iL*_) is the set of the below link nodes of the left hand side of a production of *p*_*i*_.

**Definition 25** A host graph G, a set of productions P, the formalism is PiL: = PiR, *v*_*i*_ ∈ *G*,*v*_*j*_ ∈ *SetofupNode*(*p*_*iL*_), if *Matched*(*v*_*i*,_ *v*_*j*_) = Y, then *p*_*i*_ is the candidate production of *v*_*i*_.

### Instructions

1. With the passage of the current judgment node, the current candidate production will become the target production and will produce a new candidate production.
2. The current judged node may have multilayer target productions; the target production of the first judged node of the given graph is the same as the candidate production.
3. Because the method of the current judged node looking for the candidate node is compared with the upper link node of the right-hand side of the productions, the candidate node of the current judged node is the upper link node; however, the upper link node is not completely equal to the candidate node, i.e., the current candidate node is only one of the upper link nodes.

### Description of the algorithm

The basic idea of this algorithm is as follows:

1. In order to determine whether a node has matched success, it needs to match the node with the target node of the target production. At the same time, it needs to look for the candidate production in the set of productions.

The method of looking for the candidate productions involves comparing the current judged node with the upper node of the productions. If they are matched, the production is the candidate production (there may be more than one possible candidate production or there may be none).

2. The target production and the candidate productions are stored with the stacks.

3. If the current judged node is matched with the target node, the judgment continues. If they are not matched directly, then select the current candidate production to judge whether they are matched with the target node after several steps of parsing (at this time, the candidate production is the target production).

4. If a node has not matched success until the all candidate productions are used, the process backs up. If it cannot find a candidate production until it gets to the bottom of the stack, it can judge that the initial graph cannot parse and that it is not a language of the graph grammar.

This algorithm can improve the efficiency of the parsing because of the following aspects:

1. It avoids the judgment of the graph isomorphism;
2. It reduces the times of the backing up;
3. It is more targeted on the choice of productions;
4. In the process of parsing, it retains the information of the nodes and the edges that have matched successfully.

### Analysis of the algorithm

The top-down parsing algorithm is as follows:

Char UpDownParsing (G, P)

 {

  ZeroUpInterfaceNode = TraverseGraph (G);

 ZeroDownInterfacePNode = TraverseProductions (P);

 For (G.Component)

  {

   UpInterfaceMatched = UpInterfaceMatched

   (G.Component.UpInterface,p.Component.UpInterface);

  NodeMatched = NodeMatched

   (G.Component.Node,p.Component.node);

  DownInterfaceMatched = DownInterfaceMatched

   (G.Component.DownInterface,p.Component.DownInterface);

  }

 }

Instructions:

The function of TraverseGraph (G) is to traverse the initial graph G, record the label, the upper interface number and the down interface number of all the nodes, and then find out the node whose upper interface number is zero.

The function of TraverseProductions (P) is to traverse all the productions, record the label, the upper interface number and the down interface number of the nodes of each production, record the number of the link upper interface and the number of the link down interface, and then find out the node where the number of the upper interface is zero and the corresponding production.

The parsing algorithm can be described as follows:

Traverse the given graph, find out the node where the in-degree is zero, and make the node the current node; according to the production that contains the initial sign, look for the start node in the right hand side of the production, and make the production the target production and the start node the target node. If no node meets the condition in the host graph, it can determine that the given graph cannot be parsed; otherwise, make the matching judgment for the node, including the upper interfaces, the node itself, and the down interfaces.

The function of UpInterfaceMatched (G.Component.UpInterface,p.Component.UpInterface) is to make a matching judgment for the upper interfaces of the nodes that are in the given graph and in accordance with the nodes that are in the target production.

Char InEdgeMatched (G.Node,p.Node)

 {for (G.Node.InEdge)

  {for (p.Node.InEdge)

    {if ((Label(G.Node) = = Label(p.Node)) and

    (p.Node.Dangling = Y))

    Matched (G.Node,p.Node) = Y;

   if (Matched (G.Node,p.Node) = = Y)

     {G.Node.matched = Y;

    p.Node.matched = Y;}

   }

  }

 }

The function of NodeMatched (G.Component.Node,p.Component.Node) is to make a matching judgment for the node that is in the given graph and the corresponding node in the target production.

Char NodeMatched (G.Component.Node, p.Component.Node)

  {if ((G.Component.Node.Label = = p.Component.Node.Label) and

  (G.Component.Node.In = = p.Component.Node.In) and

  (G.Component.Node.Out = = p.Component.Node.Out))

   {G.Component.Node.matched = Y;

  p.Component.Node.matched = Y;}

 }

The function of DownInterfaceMatched (G. Node,p.Node) is to make a matching judgment for the down-interfaces of the node that is in the given graph and the down-interfaces of the corresponding node in the target production.

Char DownInterfaceMatched (G.Node,p.Node)

  {for (G.Node.OutEdge)

   {for (p.Node.OutEdge)

    {if ((G.Node.Label = = p.Node.Label) or

    (p.Node.Dangling = = Y))

    Matched (G.Node,p.Node) = Y;

   If (Matched (G.Node,p.Node) = = Y)

     {G.Node.OutEdge.matched = Y;

    p.Node.OutEdge.matched = Y;}

   }

  }

 }

### Instruction of the correctness

(1) Each node in the given graph is accessible.

The given graph is connected, and there are three types of nodes in the graph, which respectively satisfy: Node.In = 0 or Node.Out = 0 or (Node.In! = 0 and Node. Out! = 0); a node that satisfies Node.In = 0 is the start node, the one that satisfies Node.Out = 0 is the final node in the parsing algorithm, and the in-degree of the nodes that satisfy Node.In! = 0 and Node.Out! = 0 is not zero; so start from the node where the in-degree is zero to make matching judgment, and then every node is accessible.

(2) There are matched target nodes for each node in the given graph.

Starting from the first node where the in-degree is zero, it can find out the target productions, and with the passage of nodes, each node can find out the corresponding target node. If a node is not matched successfully with the corresponding target node, it can look for the candidate productions in the set of productions and then choose a candidate production as the current target production; otherwise, the next node continues.

(3) The matching judgment for each node is effective.

The component is the basic element; it includes a node, the upper interfaces and the down interfaces. A component is matched successfully only if all the three aspects are matched successfully.

(4) Each recursive call in the algorithm is effective.

Actually, the algorithm is a recursive one; it uses the left-hand side of the target production embedding into the host graph and needs to look for a sub-graph where all nodes are matched with the corresponding nodes in the host graph. For each node, the matching has two scenarios: one is matched with the target node success, and the other is that which starts from the current judgment node looking for the candidate production in the set of the productions and then continues the matching judgment.

(5) Every call can be ended, so the algorithm can be ended.

Every recursive process involves the matching judgment for the nodes; the judgment has a return value, yes or no, and the number of the node of the right hand side of the target production. So, the recursive process either stops due to a node or an edge matched fault or the recursive call is a success and backs up to the upper story. Therefore, every call can be ended, and thus, the algorithm can be ended.

According to the description of the algorithm, we analyze its time complexity and try to determine the specific factors that affect time complexity, which should therefore reduce the time complexity.

Set the number of the nodes of the given graph as n, the number of the productions as s, the largest number of the candidate production for each node as m, the largest in-degree of the nodes in the productions as u, the largest out-degree as v, and the largest number of the nodes in the right hand side of every productions as p.

The following steps describe the time complexity:

Step 1: the time-complexity of traversing the given graph is *O*(*n*+*e*); e is the number of the edges of the given graph;
Step 2: the largest time-complexity is *O*(*s)*;
Step 3: the largest time-complexity is *O*(*u*!+*n*·*m*·*v*!);
Step 4: the largest time-complexity is *O*(*s*+*m*+*n*·*m*·*v*!);
Step 5: the largest time-complexity is *O*(*n*·*m*·*v*!);

So, the total time-complexity is:

 T=O(n+e)+O(s)+N(0(u!+n⋅m⋅v!)+O(s+m+n⋅m⋅v!) +O(n⋅m⋅v!) = O((n+e+s)+N(u!+n⋅m⋅v!+s))

From the definition of time-complexity above, we can see that it mainly depends on N. N is not the number of the nodes in the given graph but, rather, the number of times that they have been disposed in the parsing process.

For a node i, the number of times is determined by three factors:

1. The visited numbers by the nodes before the node i;
2. The matched numbers of the node i with the target node;
3. The visited numbers by the nodes after the node i.

The visited numbers by the nodes before the node i depend on the visited numbers for the direct former node of the node i and the visited numbers of the former node for the node i:

N i1 = u (m+1) Ni-1

The matched times between the node i itself and the target node is:

N i2 = m+1

The visited times between the node i and the later nodes is:

N i3 = v (m+1)

So, the visited times of the node i is:

N i = N i1+N i2+N i3 = u (m+1) Ni-1+ (v+1) (m+1)

Because the first node does not have former nodes,N1 = v+1

For a convenient description, we set a = u(m+1), b = (v+1)(m+1), and then

Ni = aNi-1+b

If we suppose $\text{c}=\frac{\text{b}}{\text{a-1}}$, then N_i_ + c = a(N_i-1_ + c); we can see that the progression N_i_ + c is a geometric progression; noting that ${\text{M}}_{\text{1}}={\text{N}}_{\text{1}}+\text{c}=\text{v}+\text{1}+\text{c,q}=\text{a}=\frac{{\text{M}}_{\text{i}}}{{\text{M}}_{\text{i-1}}}=\text{ u(m}+\text{1)}$, we get: $\sum_{i=1}^{n}M_{i}=\frac{M_{1}-M_{1}\cdot q^{n-1}}{1-q}=\frac{\left(v+1+\frac{\left(v+1)(m+1\right)}{u(m+1)-1})\cdot {\left(u(m+1)\right)}^{n-1}-(v+1+\frac{\left(v+1)(m+1\right)}{u(m+1)-1}\right)}{u(m+1)-1}$

Suppose *w* = *u*(*m*+1)-1,*k* = (*v*+1)(*m*+1),

Then,

$$\sum_{i=1}^{n}M_{i}=\frac{\left(v+1+w){\left(w+1\right)}^{n-1}-(v+1+\frac{k}{w}\right)}{w}$$

So, $N=\sum_{i=1}^{n}M_{i}-n\cdot \frac{k}{w}=\frac{(v+1+w){\left(w+1\right)}^{n-1}-(v+1+\frac{k}{w})-nk}{w}$

$$\begin{array}{l} T=O((n+e+s)+\text{N(u!}+\text{n}\cdot \text{m}\cdot \text{v!}+\text{s)})\\ =O(\frac{\text{(u!}+\text{n}\cdot \text{m}\cdot \text{v!}+\text{s)}(v+1+w){\left(w+1\right)}^{n-1}}{w}) \end{array}$$

We can see that the main factors influencing the time-complexity are the number of the nodes in the given graph (n), the number of the candidate productions (m), the in-degree and the out-degree of the nodes (u and v). Among them, the n is the key factor, as it decides the scale of the problem; m, u and v are also important factors, as they are the main reason for recall. The given graph comes from the application requirements. Once it is ascertained, the largest in-degree and the biggest out-degree are ascertained.

### Comparation with the other graph grammars

LGG through defining the left graph “less than” the right graph to ensure the graph grammar can be parsed. In the parsing process [16], the redex is retained, and the rest of the left graph except the context is put into the host graph. The parsing process is divided into two stages of the bottom-up and top-down. In the bottom-up stage, every step of the parsing need note the used production and the form of before and after the parsing, until the new redex can not find out in the host graph. In the stage, if it can arrive to the initial graph, the top-down operation can be proceed, and it need to create the application order of the productions used in the previous stage according with the dependency of these productions.

Because of the parsing algorithm involves two stages, the first stage need to design the special algorithm, and the second stage need to judge the dependence of the productions, thus the time complexity of the algorithm is increased.

RGG adopt the selection-free parsing algorithm when the productions meet the condition of selection-free. The algorithm does not need to re-back, the time complexity is polynomial. But the condition is not be met, it also adopt the algorithm with re-back. Zeng propose the RGG+[17] through improve on upon RGG, and give a parsing algorithm that is independent of the condition of selection-free, but the time complexity is improved greatly.

Zhu proposed the improved algorithm for EGG, through the optimization of algorithm, its time complexity is also exponential.

In conclusion, the typical algorithms of the context-sensitive graph grammars all adopt the bottom-up method, the time complexity is *O*(n^n^), where n is the number of the nodes in the host graph.

CGG is put forward in this paper, and it adopt the top-down parsing algorithm, the time complexity is also exponential, but through the analysis, we can find that the base part no longer depends on the node number of the host graph, but on the form of the productions. We know, for a grammar, the production should be as simple as possible, and the derivation or parsing operation can be completed through constants iterative use for the productions. So, the number of the largest out-edge, the largest in-edge and the node number of the productions is far less than these of host graph.

## Case Study

**Fig 11:**
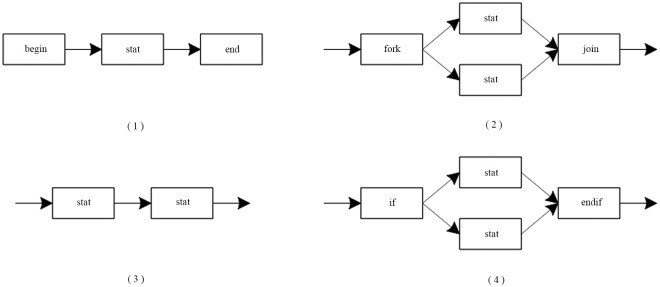
A program flow chart.

Below in the form of a chart descript the steps of the parsing for the program flow chart in Fig 11 using the productions in Fig 8. The Table 1 contains the current judgment node, the state of the host graph, the change of the target production and the target node, and lists the matching condition of the candidate production of the current judgment node and each step, and indicates the re-back condition.

**Table 1:** The parsing process of a program flow chart.

	The condition of the host graph	The current judgment node	The target production	The target node	The candidate production	The corresponding relation of the edges	The matching condition
1	(1)	begin	(1)	begin		h1<->po1	success
2	(2)	fork	(1)	stat	(3)		fail
3			(3)	fork		h2<->pt1;h3<->pt2;h3	success
4						h1<->pt1	success
5	(3)	if	(3)	stat	(5)		fail
6			(5)	if		h4<->pf1;h5<->pf2	success
7						h4<->pf1	success
8	(4)	assign	(5)	stat	(2)		fail
9			(2)	assign		h6<->2	success/parsing
10	(5)	stat	(5)	stat	(4)	h6<->pf3	success
11	(6)	endif	(5)	endif			fail /re-back
12	(7)					h5<->pf2	success
13	(8)	stat	(5)	stat	(4)	h7<->pf4	success
14	(9)	endif	(5)	endif		h8<->2	success/parsing
15	(10)	stat	(3)	stat	(4)		success
16	(11)	join	(3)	join			fail /re-back
	(12)	stat	(4)	stat			success
	(13)	join	(4)	stat			fail
17						h3<->pt2	success
18	(14)	assign	(3)	stat	(2)		fail
19			(2)	assign		h9<->2	success/parsing
20	(15)	stat	(3)	stat	(4)		success
21	(16)	stat	(3)	join	(4)		fail /re-back
		stat	(4)	stat			success
	(17)	join	(4)	stat			fail /re-back
22	(18)	stat	(4)	stat	(4)		success
23	(19)	stat	(4)	stat	(4)		success/parsing
24	(20)	stat	(3)	stat	(4)		success
25	(21)	join	(3)	join			success/parsing
26	(22)	stat	(1)	stat	(4)		success
27	(23)	end	(1)	end			success /parsing
28	(24)	λ					success

**Fig 12:**
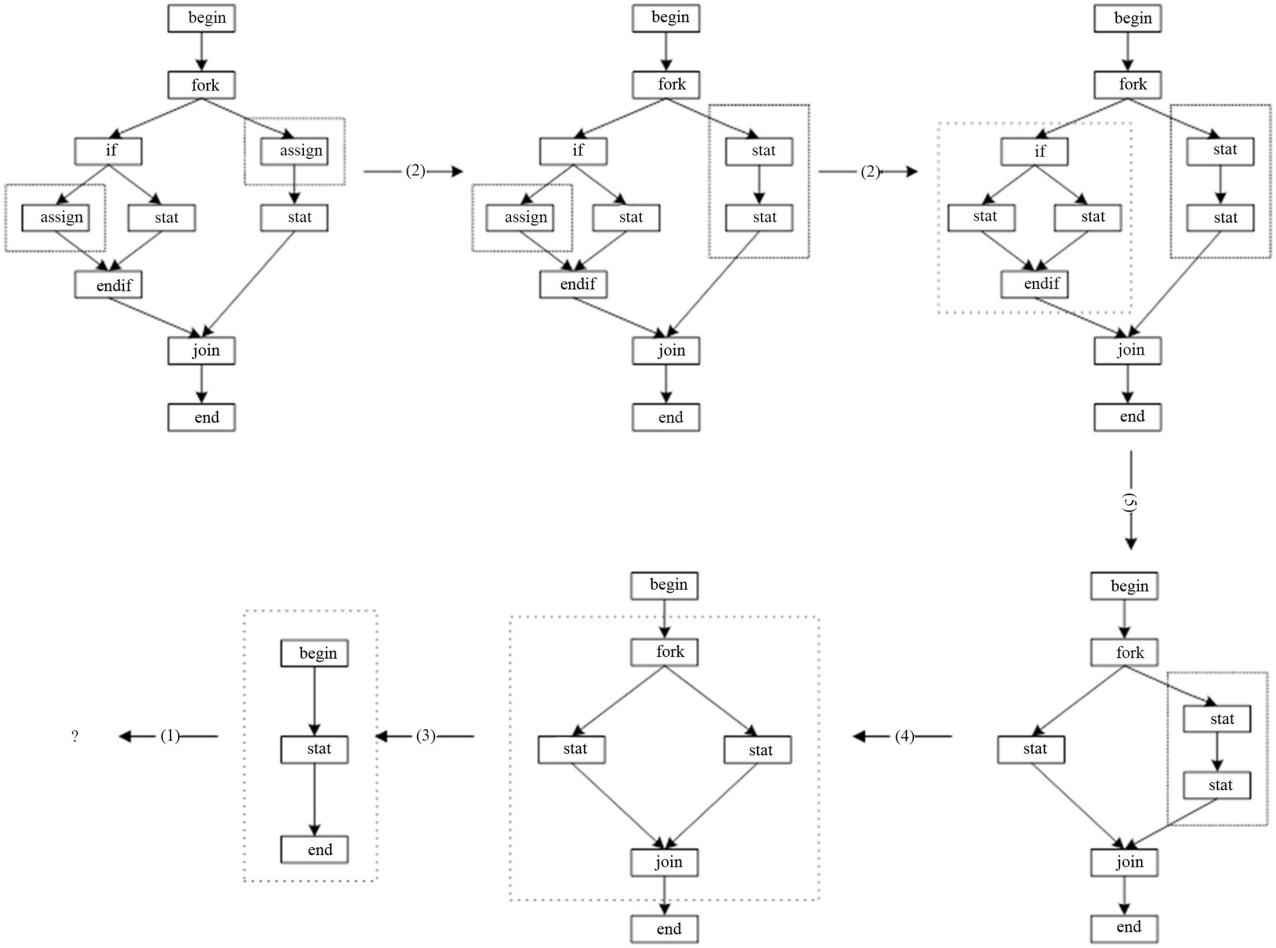
The parsing process of the program flow chart using the bottom-up method.

The traditional parsing process is as Fig 12 using the productions of Fig 8.

From the parsing processes of the two parsing methods, we can see:

1. Using the two methods, we all can get the sample result, that is to say, we can get the result whether the host graph is the language of the graph grammar.
2. Using the bottom-up method, in each step, the judgment for the graph isomorphism is need, because we must to find out the isomorphic graph in the host graph for parsing. But using the top-down method [S1 Fig], in the parsing process, the judgment for the graph isomorphism is not need.
3. Using the bottom-up method, when looking for the index in the host graph every time, the all productions must be put into. That is to say, in the host graph, we need to look for the all sub-graph that perhaps isomorphic with the each right-graph of the productions. But using the top-down method, when looking for the needed candidate productions, it is only to compare the current node in the host graph with the first node in the right graph of the each production.

To summarize the above two methods, we can see:

(1) The main operations of the top-down paring process include the nodes matching judgment, the edges matching judgment (including the judgment of the in-edges and out-edges) and the replace operation. Although the nodes and the edges that need to judge is more, but the judgment operation itself is simple, and the target node is relatively easy to find out.

The main operations of the bottom-up paring process include looking for the indexes (including the choice of the productions), subgraph isomorphism judgment and the replace operation. Among them, looking for the indexes is blind, it need to put in the all productions to look for the all subgraph which is isomorphism with the right graph of each production, the isomorphism algorithm itself is very complex.

(2) The replace operation of the two parsing algorithms is the same.

(3) For the case of the re-back, due to the looking for the target node is targeted for the top-down parsing method, so the mainly cases of the re-back are the multiple target node and (or) the matching judgment between the edges. If it can make the up-nodes are not same when the productions are designed, it can reduce the possibility of the re-back. The matching between the edges includes the number of the edges and the label of the edges, which operation is simple.

Using the bottom-up parsing method, the parsing process needs to constantly look for the indexes. When the multiple indexes are found out and through a index is not to pars successfully, it need to re-back to look for the indexes again, so the time-complexity is high.

So the top-down method can improve the efficiency of the parsing because of the following aspects:

1. It avoids the judgment of the graph isomorphism;
2. It reduces the times of the re-back;
3. It is more targeted on the choice of productions;
4. In the process of parsing, it retains the information of the nodes and the edges that have matched successfully.

## Looking Forward

In order to reduce the time-complexity further, while designing the productions, the following aspects should be considered:

1. According to the rule of selecting the candidate productions, to reduce the candidate productions, the above link nodes in the productions should be disaffiliated.
2. We call a candidate production s an invalid production if the current judgment node is not parsing to the target node through the candidate production. In order to prevent the use of these invalid candidate productions, the productions can be preprocessed before the parsing by building the parsing trees.

## Supporting Information

S1 Fig The parsing process of the program flow chart using the top-down method. (TIF)
