# Supplementary figures and images for: Research on the Top-Down Parsing Method for Context-Sensitive Graph Grammars
Source: PLoS One. 2015 Nov 30;10(11):e0142776. doi: 10.1371/journal.pone.0142776 (PMC4664237; doi:10.1371/journal.pone.0142776)

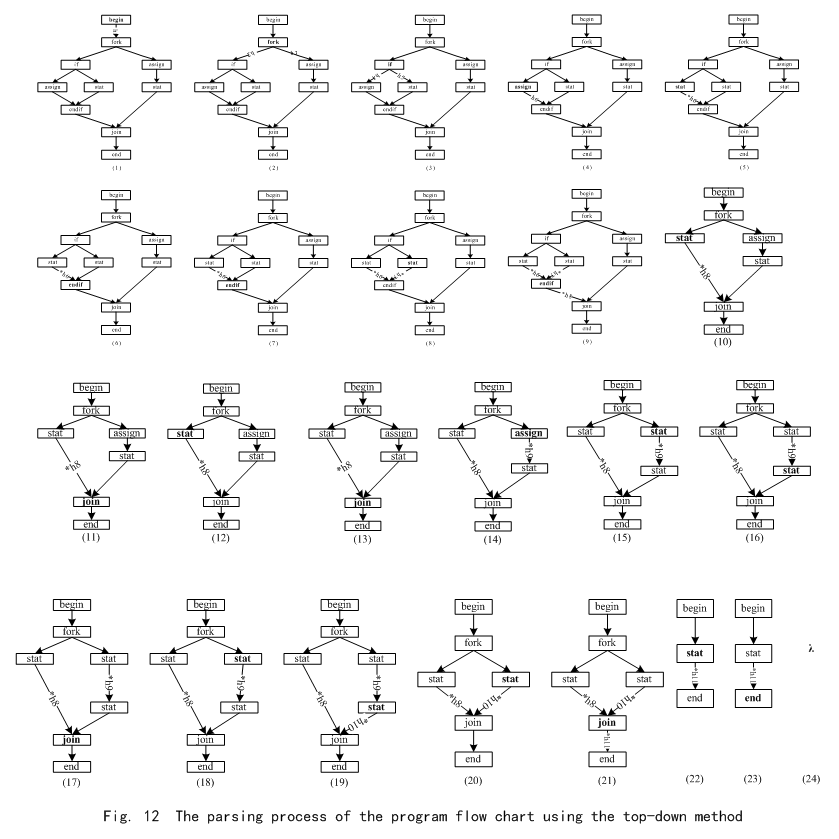

Supplement: S1 Fig — (TIF) [file pone.0142776.s001.tif]
